# A Case in Practice

**Published:** 1885-05

**Authors:** H. H. Edwards

**Affiliations:** Madrid, Spain.


ARTICLE V.

### A CASE IN PRACTICE.

(Death of the Pulp in a Perfectly Sound Tooth from an Unknown Cause.)

BY H. H. EDWARDS, D. D. S., MADRID, SPAIN.

Description and habits of patient:—

Age, 29 years; married; of a nervo-bilious temperament; very regular habits; a smoker; takes a fair amount of fresh air daily; health in general, good, though not of robust constitution; slight build; brown hair; blue eyes; twenty-seven sound teeth standing, one tooth the pulp of which—the trouble in question—is dead; the two upper first molars and the right lower first molar were extracted when a youth; the third left lower molar not yet erupted. He had regular good health until five years ago, when rheumatism intervened, resulting in fever. During the last five years has lived in England, United States of America, France and Spain, in which latter country he now is, having been located there for two years. Teeth, strong, dense and upper incisors naturally separated. Profession, dentist.

History of the tooth in question:—

The left upper lateral incisor. Originally it articulated inside the left lower cuspid; at 12 years of age it was brought outside, and into its proper line. It was always a little longer than the central incisors and lay in position slightly oblique from the median line. Until date the patient never had any suffering whatever in said tooth. Towards the tenth day of intense pain it became slightly loose and elongated.

Circumstances attending the first paroxysms:—

About six weeks ago, while at breakfast, he happened to place the tip of the tongue against the lingual portion of the festoon of the gum, and experienced such an intense

—as it appeared to him—excruciating nerve-pain, that, until examination proved to the contrary, he believed it must have arisen from an exposed pulp beneath the margin of the gum. Such was the character of the pain.

The appearance and condition of the gum:—

Both lingually and labially the gum appeared perfectly normal. Throughout the duration of pain the labial surface never presented hyper-sensibility, while, on the contrary, lingually it was acutely sensitive, though unattended by any redness or swelling.

Progress and duration of pain:—

The progress was an upward one, gradually augmenting until the patient was driven by it to undergo the operation.

The duration of the pain was twelve days, only ended by the operation.

During the first few days the tooth ached when the tip of the tongue or any food in mastication was brought in contact with the lingual surface of the gum, or by the contact of anything hot with the tooth itself. Pressure did not affect it. About the sixth or seventh days a peculiar "welling up" pain recurred at intervals, as if the nerves of the pulp exhausted themselves, and after a rest of a half hour or so regained fresh energy or sensitiveness; these attacks were absent during the night. On the tenth, and especially the eleventh days, these paroxysms became more frequent aud so intense as to become unbearable; they would commence with a slightly itching pain and within a minute would assume such a full, bursting pain as though the tooth must split unless some relief offered. The pain confined itself to the left side, never passing the median line, and embraced every tooth, especially the one in contact—the left lower cuspid—and extended to the left temple.

Remedies applied:—

As it is proverbial that shoemakers wear the worst shoes, etc., etc., so dentists as a rule, when in full practice, in the same ratio neglect their own teeth. Simply through want of time, and looking forward to the vacation when their teeth can be thoroughly attended to, they are apt to put off the evil day. In this case it was so, and the only remedy he tried that had the slightest effect in calming the agony was cold water, which, the moment it was brought in contact with the offending tooth, caused the pain gradually to decline and for a period to entirely disappear. Direct upward pressure would to a degree alleviate the pain, but lateral pressure aggravated it.

Ultimate results:—

On the twelfth day he noticed that the tooth had turned a purple-grey color, so then sought professional aid and had the pulp-chamber drilled into and opened up from the lingual face. The pulp was found congested, and was exceedingly painful during the operation of cleaning the canal. Chloride of zinc was injected, and so it remained for two days, when the root was filled with cedar-wood, dipped in plastic oxy-chloride of zinc—which root-filling, by the way, I have tested, and have several on a two-year record doing well—then when the soreness had passed off, the coronal cavity was plugged with gold.

Prognosis:—

At the present moment the tooth is firm and has not given the slightest trouble, being nearly a month since the operation. I thoroughly believe, though perhaps contrary to the theory of Dr. Sexton, that it will subserve its purpose and do good work for many years to come. Of course its fate will eventually be the fate of all so-called "dead' teeth, but I think we should be rejoiced to know that we have saved one more tooth from the immediate fatal clutch of the forcep.

What caused the death of the pulp ?

1. Dental caries? No.
2. A blow? No.
3. Excessive heat or cold in contact with tooth? No.
4. Cracks in the enamel? No.
5. Pulp nodules? No.
6. Reflex irritability from functional derangement of the stomach? No.

7. Atmospheric changes? Possibly.

8. The effects of rheumatism? Probably.

9. Reflex irritability from non-erupting lower third molar? Possibly.

10. The effect of having been regulated? Query.

11. Peridental irritation? I think not.

The foregoing diagnosis reduces the lesion to five possible causes.

Observations and enquiry:—

The case was certainly one of acute pulpitis, which by neglecting remedial means ran on to chronic pulpitis, and, no doubt, by the frequent use of cold water—not making it "persistent"—hastened on congestion. The pain was first felt around the lingual portion of the gum, thereby suggesting peridental trouble through mechanical or chemical injury. Upon examination for any cause, as, for instance, a fish-bone or bristle from tooth brush, etc., made almost immediately, no trace of such was found. I have in my own mind dismissed periodontitis as being the cause, because the pain, in the first place was only felt on the one side, and there was perfect exemption from pain—upon pressure—upon the other. Also, I doubt if acute periodontitis could exist for ten days without a loosening or elongation of the tooth taking place. That the pulpitis produced periodontitis I have no doubt whatever, because, on the last three days the tooth actually was loose and elongated, which state continued during the two days after the root was filled.

The influence of atmospheric changes—and I may couple with that the fact of having caught cold in the tooth—may, I think, be also dismissed, on the ground that, although the winter here has been exceptionally severe, with great variations in thermometer and barometer, still, the worst changes were past at the time of the trouble. On account of the sudden and persevering character of the attack, I rather attribute it to some local or systemic cause.

Let us see how the "Reflex irritability from a non-erupting lower third molar" fits. The molar is on the same side of the median line, at least, I believe it is, for there being no indication of its presence, I conclude it ought to be—and no inconvenience whatever has been experienced. Again, by the sudden and persevering character of the attack, I can understand a non-erupting tooth causing trouble either to its fellow tooth or teeth, in its effort to erupt, or even to produce a chronic state of things elsewhere; but if the lesion was brought about by a state of chronicity, I take it that pain, or at least uneasiness, would have given some warning weeks or perhaps months beforehand.

Well, let us try and fix rheumatism as the cause producing the lesion. Dr. Garretson says in his Oral Surgery, page 361, paragraph 1, "In a constitutional direction, rheumatism is, perhaps, the most frequent source." He is speaking of teeth, perfect or seemingly so, but he does not state how such a state of things is brought about, neither does he give the characteristic symptoms arising from such a cause. Of course one can understand the effect of rheumatism, as in this case, covering a period of four or five years, depressing the nervous system at large, and no doubt weakening the heart's action; but why, I must repeat, the sudden and perserving character of the attack, after so long a period? Why that particular tooth and no other? It is the pathological tooth, I admit, but chronicity, on this supposition, would surely be established, and a warning given before death of the pulp could take place.

Lastly, I would ask my professional brethren, through the indulgent means of the *Dental Practitioner*, whether they have on record any teeth that have been regulated—not after twelve years of age—giving trouble through the mechanical interference with nature's work? Or is it possible, in the course of being pushed out at a sharpish angle, so to twist the apical portion of such a tooth that the passage for the nerves and blood-vessels of the pulp has become constringed, thereby creating a state of affairs mischievous enough at any moment to strangulate the vessels and so cause a rapid death of the pulp? In this case the exploring probe showed the apical portion of the root to be so bent. I may add that two experienced members of the profession have examined the tooth but have not been able to diagnose the cause, though they have treated similar teeth in their practices. Two of such instances I have lately seen, being the two lower central incisors in the same mouth.

What I want to know is, with all the above history at hand from which to deduct facts, what should cause the pulp of an apparently unblemished tooth to take on active inflammation and to die after a good record, say twenty years.—*The Dental Practitioner.*

